# Mechanical Properties of Eco-Friendly, Lightweight Flax and Hybrid Basalt/Flax Foam Core Sandwich Panels

**DOI:** 10.3390/ma17153842

**Published:** 2024-08-02

**Authors:** Marzena Pawlik, Urvashi Gunputh, Daniel Odiyi, Sarah Odofin, Huirong Le, Paul Wood, Angelo Maligno, Yiling Lu

**Affiliations:** 1 School of Engineering, College of Science and Engineering, University of Derby, Markeaton Street, Derby DE22 3AW, UK; d.odiyi@derby.ac.uk (D.O.); y.lu@derby.ac.uk (Y.L.); 2 Institute for Innovation in Sustainable Engineering, University of Derby, Quaker Way, Derby DE1 3HD, UK; p.wood7@derby.ac.uk (P.W.); a.maligno@derby.ac.uk (A.M.); 3 Department for Business, Energy, and Industrial Strategy (BEIS), Teddington TW11 0LW, UK; sarahodofin@gmail.com; 4 The Future Lab, Tsinghua University, Beijing 100084, China; lehr@tsinghua.edu.cn

**Keywords:** natural fibre, nonwoven flax fibre skin, woven flax/basalt fibre skin, bio-sandwich, mechanical properties, core shear test, flexural testing, edgewise compression

## Abstract

Greener materials, particularly in sandwich panels, are in increasing demand in the transportation and building sectors to reduce environmental impacts. This shift is driven by strict environmental legislation and the need to reduce material costs and fuel consumption, necessitating the utilisation of more sustainable components in the transportation and construction sectors, with improved load-bearing capabilities and diminished ecological footprints. Therefore, this study aims to analyse and evaluate the structural performance of polyethylene terephthalate (PET) core and flax or basalt/flax FRP sandwich panels as an alternative to conventional synthetic materials. The novel eco-friendly sandwich panels were manufactured using the co-curing technique. Four-point bending, edgewise compression and core shear tests were performed and insights into how the skin properties affect the strength, stiffness and failure mode of specimens were provided. The stress–strain behaviour, facing modulus and strength, flexural rigidity, core shear strength and failure modes were evaluated. The flexural facing modulus of the flax and flax/basalt sandwich skins were found to be 5.1 GPa and 9.8 GPa, respectively. The flexural rigidity of the eco-friendly sandwich panel was compared with published results and demonstrated a promising structural performance. The environmental benefits and challenges were outlined and critically evaluated focusing on transportation and construction applications.

## 1. Introduction

Owing to their low weight and high structural rigidity, sandwich panels are commonly used in transportation and construction applications. These panels are composed of low-density, thick core encased by thin face sheets, namely skins, of strong and stiff material. Skins are usually made of synthetic fibre-reinforced polymers (FRPs), such as glass (GF) or carbon (CF), and present excellent mechanical performances. Thick and low-density cores promote higher bending strengths while ensuring a lightweight structure. Most conventional sandwich panels are based on honeycomb or polymeric foam cores. Synthetic skins and cores provide excellent structural properties; however, their sustainability presents a significant challenge. Due to constant global demand and more restrictive legislation to reduce the environmental footprint of structures, there is a need to design and develop more eco-friendly sandwich panels. Despite their good mechanical properties, traditional materials such as fossil-based polymers and synthetic fibres have high environmental impacts and are challenging to recycle, thus making them unsuitable for eco-friendly sandwich panels [1]. Therefore, some alternatives composed of more environmentally friendly constituents, i.e., natural fibres, a bio-derived resin system and recycled cores, are explored here [2,3,4,5]. In the following Section 1.1, Section 1.2 and Section 1.3, the examples, advantages and challenges of the environmentally friendly materials used as skin, cores and structures are introduced.

### 1.1. Natural Fibre Reinforced Bio-Sandwich Skin

Over the last decade, natural fibre-reinforced polymers (NFRPs) have become increasingly attractive and started to replace conventional glass fibre reinforced polymers (GFRPs). Compared to synthetic fibres, natural fibres are lighter, easier to machine, recyclable and renewable, which contribute to low energy production, environmental benefits and biodegradable systems [6,7,8,9,10]. Natural fibres are classified according to their source of origin into the three following categories: animal, plant, and mineral [11,12]. Plant fibres, the main source of natural fibres, are divided into five main types as follows: bast, leaf, seed, grass stem and wood. Among plant fibres, flax, jute and hemp [13,14,15,16,17,18,19] have gained substantial interest as reinforcements for NFRPs. In comparison to synthetic counterparts, natural fibres have a lower density but comparable mechanical properties which make them an excellent candidate for reinforcing polymeric skins (see Table 1). For example, the density of flax is about 40% less than that of traditional E-glass while having a comparable Young’s modulus and tensile strength. The tensile strength of flax fibres ranges from 345 to 1040 MPa, and the elastic modulus of flax fibres varies up to 80 GPa, which is slightly higher than synthetic E-glass fibres. More recently, mineral fibres, namely basalt have been increasingly used in NFRP due to their high tensile strength (up to 4840 MPa), and high elastic modulus (96 GPa). Along with environmentally friendly features, basalt fibres offer excellent high-temperature, light and corrosion resistance [20].

Considering the matrices present in NFRPs, thermoplastic materials (i.e., polypropylene (PP), polyethylene, and polyvinyl chloride (PVC)) are the most commonly used, while phenolic, epoxy and polyester resins are the more generally used thermosetting matrices [21]. Due to limited petroleum resources and environmental issues, researchers are constantly searching for bio-based alternatives from renewable resources, i.e., starch, lignin, cellulose, plant oils or furfural. Comprehensive reviews of bio-based polymers can be found elsewhere [22,23,24]. For example, very recently Odiyi et al. [22] reviewed the advancements in the synthesis, manufacturing and properties of environmentally friendly bio-based Polyfurfuryl Alcohol (PFA) resin. This PFA resin is considered one of the fully bio-based resins, a dark brown organic polymer obtained by the acid-catalysed polymerization of furfuryl alcohol derived from renewable agricultural waste, i.e., sugarcane [25]. It possesses comparable mechanical properties to petroleum-based thermosets as well as remarkable thermal and chemical resistance to acids, alkalis and solvents. More recently, its commendable mechanical and thermal properties have resulted in its use in fibre-reinforced polymer composite applications. The advantages of its outstanding thermal stability and fire smoke toxicity (FST) characteristics, as well as its environmental friendliness, have enabled it to be used in transportation and construction as a viable alternative to traditional petroleum-based resins. However, its use in foam sandwich panels is still very limited and their mechanical properties and feasibility are yet to be reported.

**Table 1:** Comparison of mechanical and physical properties of natural and synthetic fibres [26,27,28].

Type of Fibres	Tensile Strength (MPa)	Young’s Modulus (GPa)	Density (g/cm^3^)	Ultimate Elongation at Break (%)
Jute	200–770	27	1.3–1.5	1.5–3.0
Flax	345–1040	28–80	1.4–1.5	1.2–3.2
Basalt	4100–4840	89–110	2.7	3.2
Hemp	690	30–70	1.3	1.5–4.0
Synthetic Glass	150–550	10	2.5	1.5–3.5
E-Glass	2000–3500	70–73	2.5–2.55	2.5–3.7
Carbon	3400–4800	230–240	1.4–1.8	1.4–1.8

### 1.2. Environmentally Friendly Cores

In addition to the bio-based skin, an eco-friendly sandwich panel requires a lightweight environmentally friendly core. The selection of the core material will depend on the application and particular requirements, i.e., fire retardancy, structural strength and low weight. One of the most popular materials used in bio-sandwich panels are cores from trees including balsa wood and cork [8]. The balsa core has the characteristics of a good strength, stiffness, and significant impact resistance and is mostly used in the transport sector. The cork core has excellent thermal insulation and damping properties due to its closed cellular structure [1]. Polymer foams derived from starch, PLA, tannin or flax oil are also used as composite sandwich cores. However, these bio-based foams often have some negative properties, including brittleness and low-temperature resistance. In addition to bio-based foams, there are thermoplastic closed-cell foams. Using these foams as the core in sandwich panels provides relevant properties, such as a high permeability, low density, noise-absorbing insulation and fire retardancy [29]. In general, most foams are derived from petroleum polyol classes and result in a significant environmental impact. However, reusing/recycling them could reduce the overall $CO_{2}$ emissions. An interesting class of foams is based on polyethylene terephthalate (PET) that can be recycled to PET particles and incorporated into different materials [29].

### 1.3. Bio-Sandwich Structure

There are many factors motivating the interest in environmentally friendly sandwich structures due to their great potential to replace synthetic sandwich panels in transport and construction applications. However, the overall behaviour of these panels depends on many factors. So far, there have been some research works that have investigated the effects of the composition of bio-sandwich panels on their flexural properties [3,4,6], impact behaviour [30], fire retardancy [31] and sound absorption [9]. Bach et al. [1] analysed the flexural and impact behaviour of a bio-sandwich panel composed of hardwood skins and mushroom foam of various thicknesses. The flexural properties were improved significantly with an increase in the thicknesses and Young’s moduli of wooden skins. Sandeghian et al. [8] compared two types of skin materials, namely glass and flax fibres and two types of cores: polypropylene honeycomb and cork core materials. It was found that the flexural stiffness and the transverse shear rigidity of a 22 mm thick cork with two layers of flax fibre skin were comparable to those of a 12 mm thick honeycomb with one layer of glass skin, thus showing the exciting potential of natural sandwich panels. Besides structural performance, bio-sandwich panels provide excellent sound absorption properties compared to synthetic-based panels. For instance, Zhang et al. [9] reported that flax–PET bio-sandwich showed around a 50% improvement in sound absorption coefficients in the range of frequencies from 4000 to 6000 Hz compared to its glass fibre–PET counterpart. Kandare et al. [32] highlighted the fire-retardant properties of flax/epoxy and balsa cork sandwich panels, whereas Monti et al. [33] developed a finite element model to study the vibration behaviour of bio-sandwich panel composed of a thermoplastic matrix, flax fibre and balsa wood core.

Although long flax fibres have previously been studied for their use in sandwich beams [4,7,8], the effects on nonwoven flax or hybrid basalt/flax bio-based skins in combination with recycled PET foam on their mechanical performance, to the best of authors’ knowledge, have yet to be reported. The skin is made of natural fibres combined with bio-based PFA resin. Previously, it has been proven that a combination of basalt and flax in hybrid composites improves the overall mechanical properties of the composites [10,34]. Therefore, this study aims to analyse and evaluate the structural performance of PET core and flax or basalt/flax FRP sandwich beams as an alternative to conventional synthetic materials used for construction and building, i.e., GFRP sandwich panels. This article provides a detailed analysis of the mechanical properties of these novel environmentally friendly sandwich structures. Four-point bending, edgewise compression and core shear tests were performed and insights into how the skin properties affect the strength, stiffness and failure mode of specimens were offered. The results were critically evaluated and compared with the published literature. The novelty of this article lies in its integration of eco-friendly materials into high-performance panels, and the detailed mechanical property analysis demonstrated the potential applications of the proposed sandwich structure for various transportation (i.e., caravan floors, walls) and building (i.e., dividing walls) applications.

## 2. Materials and Methods

**Figure 1:**
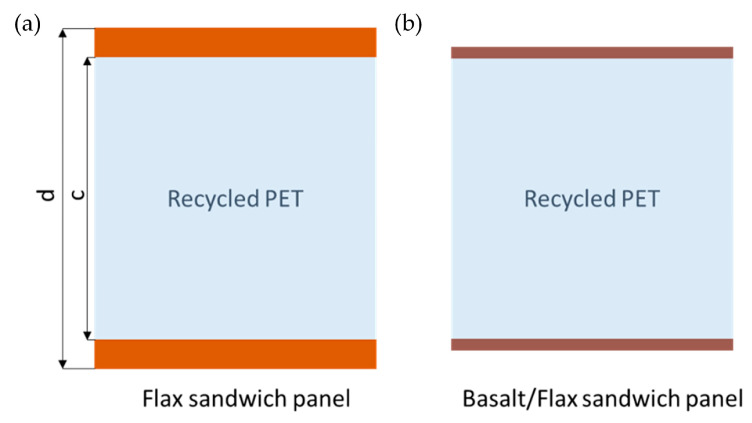
Schematic of manufactured sandwich panels: (**a**) flax; (**b**) basalt/flax sandwich panels.

Two types of fibre materials, namely flax and a combination of flax and basalt, together with recycled PET foam, were used to manufacture bio-sandwich panels, as shown in Figure 1. Four-point bending, three-point bending and edgewise compression tests were performed to evaluate the structural behaviour of the bio-sandwich panels. Section 2.1 focuses on manufacturing a flax sandwich panel, while Section 2.2 describes hybrid basalt/flax panels. Both sandwich panels were subjected to the same mechanical testing outlined in Section 2.3.

### 2.1. Flax Sandwich Panel

The flax sandwich panels were manufactured by using a co-curing technique using the hot press at Evolution Composites (Tibshelf, UK) according to the material supplier’s specifications. A prepreg of a chopped non-woven flax fibre impregnated with PFA bio-resin, Fibripreg, (EcoTechnilin, Valliquerville, France) acted as a skin. The prepreg had a density of 1600 $g/m^{3}$ and a flax volume fraction of 55%. EcoTechnilin claims that Fibripreg is one of the first 100% bio-based and fully energetically recoverable composites at the end of life, contrary to glass fibre-reinforced composites. Its use allows for weight saving in transport applications, reducing $CO_{2}$ emissions [35].

A closed-cell, thermoplastic, recyclable PET rigid foam (AIREX, London, UK) acted as a core. This type of foam is characterised by excellent fire, smoke and toxicity properties [36]. The total average thickness of the flax sandwich samples was 16.67 (±0.3) mm with an average core thickness of 13.87 (±0.29) mm and a nominal skin thickness of 1.4 mm. A schematic of the manufactured flax sandwich panel is shown in Figure 1a.

### 2.2. Basalt/Flax Sandwich Panel

**Figure 2:**
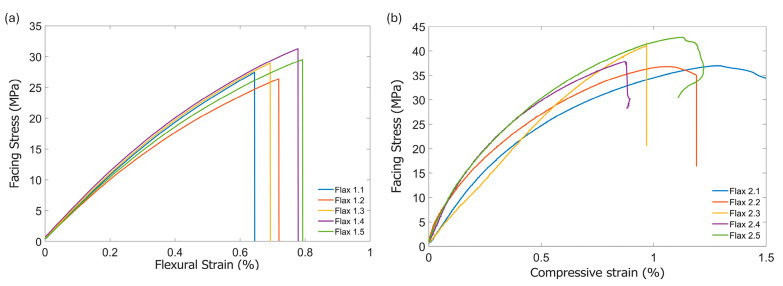
Stress–strain curve of flax sandwich panel in (**a**) long beam flexural test and (**b**) core shear test.

The basalt/flax sandwich panels were also manufactured through the co-curing method at Evolution Composites (Tibshelf, UK). The skin material was a hybrid woven basalt/flax fibre fabric pre-impregnated with PFA bio-resin (FibriRock, EcoTechnilin, France). The flax to basalt to resin weight ratio was 30:35:35 wt%. The nominal thickness of the cured skin was estimated at 0.5 mm (see Figure 2b). The same PET foam as in the flax sandwich panel was used. The average core thickness was 14.2 (±0.14) mm. The total average thickness of the flax sandwich samples was 15.36 (±0.3) mm. The difference in thickness between flax and basalt sandwich panels resulted from manufacturing parameters and the initial thickness of each prepreg layer.

### 2.3. Mechanical Testing

Mechanical testing was performed using Shimadzu AG-X Plus with a load cell range of 0 ± 100 kN, Instron with a load cell range of 5985 0 ± 250 kN and an imetrum optical extensometer (Composite Test & Evaluation Ltd., Honiton, UK). All testing was conducted at room temperature, defined as 23 ± 3 °C, and a maximum of 60% room humidity. Five specimens were evaluated for each configuration. The following static tests were performed:

- Long beam flexural test (four-point bending)

The flexural strength and facing stress of the sandwich panels were determined using equipment and methods that comply with the requirements of ASTM D7249 [37]. Specimens were loaded via V-groove loading pads instead of rollers, as recommended in ASTM D7249, to avoid damaging the thin-facing material of the sandwich panels. Testing was performed at a constant speed of 6.0 mm/min until specimen failure. Specimens with a width of 75 mm, a support span of 560 mm and a 100 mm loading span, as per the standard configuration, were tested. Facing stress, effective facing modulus, flexural rigidity and failure mode were reported. Note that flexural rigidity (*D*) is the product of the overall sandwich panel modulus and the second moment of area (Equation (1)) according to ASTM D7250 [38].

(1) $$D=\frac{E\left(d^{3}-c^{3}\right)b}{12}$$

In Equation (1), E represents effective facing modulus (MPa), *b* is the width of the panel (mm) and *c* and *d* are the core and sandwich thicknesses, respectively (mm).

- Edgewise compression

All testing was performed using equipment and methods according to ASTM C364 [39]. Specimen width and length were determined to be 50 mm and 80 mm, respectively, as per ASTM C364 to reduce the risk of global buckling, and the thickness was that of the supplied sandwich material. Testing was performed at a constant speed of 0.5 mm/min until failure. Properties reported include facing strength, effective facing modulus, facing failure strain and failure modes.

- Core Shear (three-point bending)

The core shear properties of sandwich panels were determined according to ASTM C393 [40]. The specimens with an average width of 75 mm were evaluated in a three-point bending set-up with a lower support span of 150 mm; the load was applied using 60 shore hardness rubber pads under loading blocks with V groves pivoting on 10 mm radius supports as directed by ASTM C393. Testing was performed at a constant crosshead speed of 6 mm/min. The properties reported are ultimate core shear stress along with the failure mode.

## 3. Results

### 3.1. Mechanical Properties of Flax Sandwich Panel

**Figure 3:**
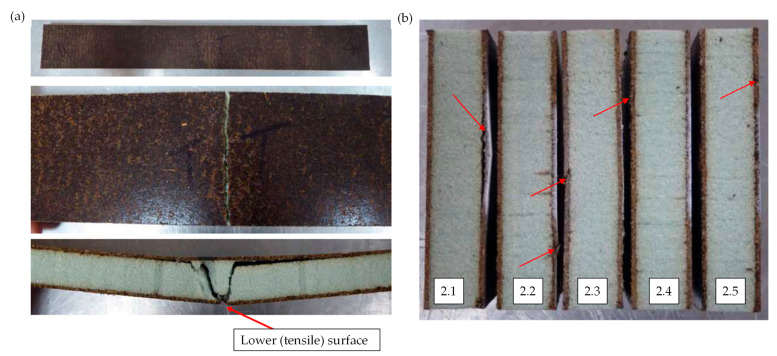
Typical flax sandwich panel failure of (**a**) long beam flexure test and (**b**) edgewise compression. Specimen labelling corresponds to the failure mode indicated in Table 3.

Figure 2a represents the facing stress–strain curves of the flax sandwich in the long beam flexural test. A fairly linear elastic response of facing stress to strain was observed. The maximum force, corresponding to the material failure, was associated with the tensile failure of the lower surface of flax/PFA skin (see Figure 3a). The effective facing modulus of the skin was 5.10 ± 0.25 GPa and the average flexural facing strength was 28.71 ± 1.89 MPa (see Table 2). The average flexural rigidity of the sandwich panel was 69.98 ± 1.27 $MNmm^{2}$. The average flexural strain at failure was 0.73 ± 0.06%. No delamination between the skin and the core occurred during the flexural test, suggesting good bonding between the skin and core. The typical failure mode of long beam flax specimen was tensile skin failure, as shown in Figure 3a.

A nonlinear compressive behaviour was observed in the flax sandwich compressive facing stress–strain curve (Figure 2b). The average compressive facing strength of the flax sandwich construction was 39.19 ± 2.82 MPa. Also, the average effective compressive facing modulus in the flax sandwich panels was 5.36 ± 0.46 GPa and they were in a similar range as long beam test measurements. The standard deviations of compressive properties were within the acceptable range and likely arose from material defects or manufacturing inconsistency. The dominant failure mode recorded during the testing was skin compression failure, as presented in Figure 3b. Specimen 2.1 experienced a combined face buckling/compression failure; however, it did not significantly affect the compressive facing strength, as depicted in Table 3.

**Figure 4:**
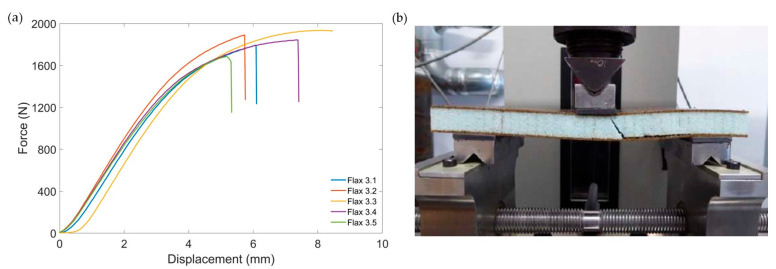
Representation of core shear flexure test of flax sandwich panel: (**a**) force–displacement curve; (**b**) typical failure mode.

Figure 4a shows force–displacement curves of the flax sandwich panel in three-point bending. The maximum force, together with sample dimensions, was used to calculate the core shear strength following the testing standard. The average core shear ultimate strength of the flax sandwich construction was found to be 0.79 ± 0.05 MPa, as shown in Table 4. The validity of the core shear strength depended on the failure mode of the tested specimen. All five specimens showed core shear failures, with three specimens also suffering from facing skin failures. Facing skin failures were considered secondary, and occurred after core shear, ensuring all measurements were valid. The typical failure mode of the core shear specimen is shown in Figure 4b.

### 3.2. Mechanical Properties of Basalt/Flax Sandwich Panel

**Figure 5:**
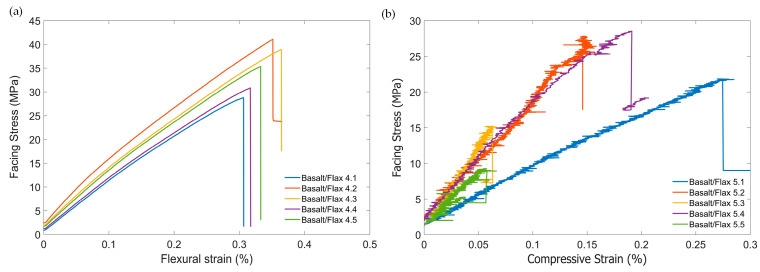
Stress–strain curve of basalt/flax sandwich panel in (**a**) long beam flexural test and (**b**) edgewise compression test.

Figure 5a illustrates the almost linear relationship of facing stress–strain for the basalt/flax sandwich panels. The average effective facing modulus of the basalt/flax sandwich panel was calculated to be 9.76 ± 0.73 GPa. The average flexural strain at failure was 0.73 ± 0.06%. The average flexural facing strength was found to be 35.01 ± 5.21 MPa. The considerable variation in the flexural strength was a consequence of insufficient resin in the facing material, resulting in a dry fabric with poor adhesion (see Table 5).

**Figure 6:**
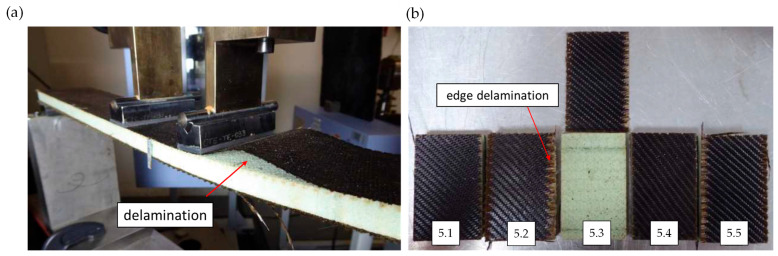
Basalt/flax sandwich panel failure of (**a**) long beam flexure test and (**b**) edgewise compression. Specimen labelling corresponds to the failure mode indicated in Table 6.

The different failure modes of the tested samples were observed as shown in Figure 6. Two out of five basalt/flax sandwich panels (specimens 4.1 and 4.5) failed due to skin delamination, as shown in Figure 6a. Two specimens failed due to facing skin compressive failures (specimens 4.2 and 4.3), and one specimen suffered facing skin delamination on the tensile surface (specimen 4.4). The specimens which exhibited delamination showed a lower facing stress. The average flexural rigidity of the basalt/flax sandwich construction was found to be 41.71 ± 3.53 $MNmm^{2}$. This represents around a 40% reduction in flexural rigidity compared to the flax sandwich panel, which is likely caused by the difference (0.9 mm) in the facing thicknesses of the two sandwich panels (see Equation (1)).

Figure 5b represents the compressive facing stress–strain relationship for the basalt/flax sandwich specimens. The average effective facing modulus in the basalt/flax sandwich construction was found to be 12.65 ±5.27 GPa, as shown in Table 6. The high amount of variation in the results was a consequence of the poor facing to core bond, and two of the five specimens failed at loads too low to calculate a facing modulus. The average compressive facing strength of the basalt sandwich construction amounted to 20.53 ± 8.29 MPa. This represents a 48% reduction in facing strength compared to the flax sandwich construction (Table 3). All samples failed in facing sheet buckling mode. During the machining of the edgewise compression specimens, edge delamination like that shown in Figure 6b occurred. This issue was exacerbated by the dryness of the skin material, as the resin content was lost to the core.

**Figure 7:**
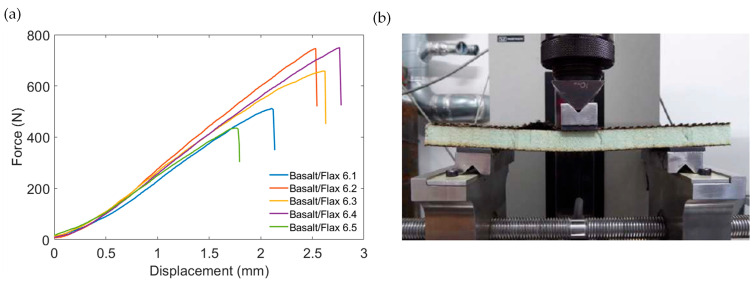
Representation of core shear flexure test of basalt/flax sandwich panel: (**a**) force–displacement curve; (**b**) typical failure mode.

Figure 7a illustrates the force–displacement behaviour of the basalt/flax panel under core shear testing. The average core shear strength of the basalt sandwich construction was found to be 0.28 ± 0.6 MPa (see Table 7). All five specimens failed due to compressive facing skin delamination, as shown in Figure 7b. This is an invalid failure mode. The insufficient core-to-skin bond and low flexural rigidity resulted in the poor shear load being transferred to the core material, allowing failures to occur. Once again, this is a symptom of there being insufficient resin in the facing skins.

## 4. Discussion

The present study has investigated the mechanical properties of two types of novel flax and basalt/flax environmentally friendly sandwich panels. The intended applications for these materials were panels and dividing walls used in transportation (i.e., caravans) determined by Evolution Composites, Tibshelf, UK. Firstly, the feasibility of manufacturing the combination of the PFA prepreg system and PET foam core was investigated. The suppliers of the PFA resin systems used (EcoTechnilin, Valliquerville, France) claimed that they were 100% bio-based. They are relatively new resin systems and lack full mechanical characterisation; therefore, at the start of the project, it was unknown how efficiently the PFA resin would bond to the PET core. A hot press was used to manufacture the sandwich panels and the PFA bio-resin adhered well to the PET foam core. It was observed that the bonding strength depended on the amount of resin in the prepreg. During sample preparation, it was observed that the basalt/flax woven prepreg contained less resin (35%) compared to the flax one (55%), which affected the bonding between the skin and core during the manufacturing process. This influenced the accuracy of the results, as delamination between the skin and core was the primary failure mode in basalt/flax sandwich panels.

Regarding the mechanical properties of the skin, it is commonly accepted that short fibre composites are characterised by having lower properties than aligned fibres. In the literature, the flexural properties of non-woven flax fibre mats can be seen to range from 1.2 to 10 GPa [16,27]. These properties depend on the volume fraction of the fibre, the fibre matrix bonding, the length of the fibres and the manufacturing conditions. For example, Habibi et al. [17] reported an increase in the flexural moduli of short flax fibre–epoxy composites from 6.94 GPa for a 20% fibre volume fraction to 9.81 GPa for a 40% fibre volume fraction. From the present analysis, the flexural modulus of flax fibre skin was found to be 5.10 GPa which is in line with published data [19] and the supplier datasheet. From the edgewise compression tests, the effective modulus of 5.36 GPa and facing strength of 39.19 MPa were found to be slightly higher than the flexural properties. The compressive facing strength of the flax fibre skin was 10.48 MPa higher than the flexural facing strength. All the samples in the flexural test failed in the tensile mode of the lower skin, suggesting that the compression strength of this flax/epoxy material is higher than its tensile strength. The measured shear strength of the PET core (0.79 MPa) was in good agreement with a shear strength of 0.94 MPa for a similar type of PET closed cell form and glass/fibre sandwich panel reported by [36].

Based on experimental results, the average flexural modulus (9.96 GPa) of the basalt/flax skin materials was found to be almost 100% higher than that of the non-woven flax mat, whereas the basalt/flax PFA skin showed a 22% increase in facing strength compared to flax/PFA skin.

This is attributed not only to the alignment of the fibres (woven fabric) but also the improved properties of the basalt fibres (see Table 1). Moreover, the basalt/flax prepreg has an overall higher fibre volume fraction and lower resin content. From edgewise compression tests, the effective modulus of 12.65 GPa was found to be slightly higher than the values of the flexural properties. However, all the samples failed due to facesheet buckling caused by poor bonding of the skin to the core. It is recommended that the amount of resin in this sandwich configuration should be increased to avoid these issues.

A direct comparison of the properties of the entire sandwich structure and the evidence from the literature is challenging, as the flexural rigidity of the structure also depends on the skin and core thickness. Table 8 presents a comparison of various bio-inspired sandwich panel properties published elsewhere. To normalise the results, the average flexural rigidity of the structure was divided by the width of the specimen and is given in Nmm. Across the range of published results, the flexural rigidity of sandwich panels varied from $5.2\times 10^{3}\;Nmm$ to $4858.0\times 10^{3}\;Nmm$. The highest flexural rigidity of $4858.0\times 10^{3}\;Nmm$ was observed for unidirectional (UD) flax fibres impregnated with bio-epoxy and bonded to corrugated cardboard [7]. This flexural rigidity is attributed to the good elastic properties of the unidirectional flax fibre skin (~20 GPa) and the much thicker corrugated cardboard core. Sadeghian et al. [8] evaluated the flexural rigidity of UD flax skin and a cork core. The achieved flexural rigidity of $900\times 10^{3}\;Nmm$ was comparable to the $925.1\times 10^{3}\;Nmm$ rigidity found in this study for the flax sandwich panel. The flexural rigidity of basalt/flax sandwich panels evaluated in the present work exceeded the reported results for the same orientation of the fibres. For example, Henao et al. [5] investigated woven glass fibre sandwich panels with a foam core and the thickness of the whole structure was more than double that of woven basalt/flax bio-panels. The reported flexural rigidity of $372.5\times 10^{3}\;Nmm$ was 50% lower than the one achieved in this work. The nonwoven flax sandwich panel can be compared to the short fibre jute/PP with PET foam core published by [6]. Even though in this work, the flax fibre nonwoven skin had a 0.6 mm lower thickness, and the flexural rigidity of the tested bio-sandwich panel was double that of jute/PP sandwich panel.

Flax and basalt/flax PFA-reinforced bio-sandwich panels are currently being discussed as promising innovative materials for use in many applications requiring high structural properties, high-temperature resistance and sound insulation. Previous research has shown that natural fibre-reinforced bio-sandwich structures exhibited improved sound insulation [9] and thermal properties [31]. The flexural properties of the novel bio-sandwich panels reported here showed comparable or even higher properties to their glass fibre-reinforced sandwich counterparts. The potential applications of bio-sandwich panels are automotive aerospace and marine sectors, for use, i.e., in interior parts, floor, and ceiling. Bio-sandwich panels can also be used in the construction industry as lightweight cladding, facades and partition walls. The unique design and surface finish of the bio-sandwich panels make them excellent candidates for use in furniture and decorations, i.e., panel doors, cabinets. The drawback of natural fibres is their higher cost compared to glass fibres. Initially, their cost was several orders of magnitude higher. Nevertheless, each year we observe a significant increase in the number of companies supplying natural fibres and prices keep decreasing. Competitive prices between natural and synthetic fibres are expected in the upcoming years. Manufacturing the prepreg with PFA resin presents some challenges. More research is needed to improve the bonding between skin and core in the basalt/flax bio-sandwich construction. This can be achieved by modifying manufacturing conditions (i.e., venting the hot press) or through the application of an extra amount of resin to ensure full impregnation and remove dry spots.

## 5. Conclusions

This study analysed and evaluated the structural performance of PET core and flax or basalt/flax FRP sandwich panels as an alternative to conventional synthetic materials. The novel eco-friendly sandwich panels were manufactured by using the co-curing technique. Four-point bending, edgewise compression and core shear tests were performed and insights into how the skin properties affect the strength, stiffness and failure mode of specimens were provided. The stress–strain behaviour, facing modulus and strength, flexural rigidity, core shear strength and failure modes were evaluated. The flexural rigidity of the eco-friendly sandwich panel was compared with published results and demonstrated promising structural performance. The following conclusion can be drawn:

- Novel configurations of sandwich panels composed of PFA bio-resin skin and PET core were successfully manufactured using a hot press.
- The effective flexural moduli of nonwoven flax and woven basalt/flax were 5.1 and 9.8 GPa, respectively.
- The nonwoven flax bio-sandwich had a 40% larger flexural stiffness (69.98 $MNmm^{2}$) than the woven basalt/flax construction (41.71 $MNmm^{2}$). This resulted from the difference in skin thickness and adhesion of skin to the core.
- The most common failure mode of the nonwoven flax bio-sandwich panel was the tensile failure of the bottom skin. The basalt/flax sandwich construction failed due to the delamination between the skin and the core caused by insufficient bonding.
- The normalised flexural rigidity agrees well with the published data and shows a comparable structural performance.
- The potential applications of these bio-sandwich panels were identified as the automotive and aerospace sectors, for use, i.e., in interior parts, floor and ceiling. Bio-sandwich panels can also be used in the construction industry as lightweight cladding, facades, and partition walls.
- Manufacturing the prepreg with PFA resin presents some challenges. More research is needed to improve the bonding between skin and core in the basalt/flax bio-sandwich construction. This can be achieved by modifying the manufacturing conditions (i.e., venting the hot press) or through the application of an extra amount of resin to ensure full impregnation and remove dry spots.

## Figures and Tables

**Table 2:** Long beam flexural test—F = flax sandwich panel.

Specimen ID	Facing Stress (MPa)	Effective Facing Modulus (GPa)	$\mathrm{Flexural}\;\mathrm{Rigidity}\;(\mathrm{EI})\;(MNmm^{2})$	Flexural Strain at Failure (%)	Failure Mode
Flax 1.1	27.43	5.25	70.40	0.64	Tensile skin failure
Flax 1.2	26.39	4.71	67.79	0.72	Tensile skin failure
Flax 1.3	28.93	5.25	70.76	0.69	Tensile skin failure
Flax 1.4	31.26	5.30	70.93	0.78	Tensile skin failure
Flax 1.5	29.52	4.99	70.03	0.79	Tensile skin failure
Average	28.71	5.10	69.98	0.73	
St.dev.	1.89	0.25	1.27	0.06	

**Table 3:** Edgewise compression—flax sandwich panel.

Specimen ID	Compressive Facing Strength (MPa)	Effective Facing Modulus (GPa)	Failure Mode
Flax 2.1	36.95	5.37	Facing Sheet Compression/Buckling Failure
Flax 2.2	36.77	4.97	Facing Sheet Compression
Flax 2.3	41.66	4.83	Facing Sheet Compression
Flax 2.4	37.80	5.92	Facing Sheet Compression
Flax 2.5	42.79	5.70	Facing Sheet Compression
Average	39.91	5.36	
St.dev.	2.82	0.46	

**Table 4:** Core shear test—flax sandwich panel.

Specimen ID	Max Force (N)	Core Shear Ultimate Strength (GPa)	Failure Mode
Flax 3.1	1793.05	0.76	Transverse Shear/Gage/Core
Flax 3.2	1891.17	0.81	Multi-mode/Gage/Various
Flax 3.3	1935.18	0.84	Multi-mode/Gage/Various
Flax 3.4	1843.91	0.80	Multi-mode/Gage/Various
Flax 3.5	1685.43	0.72	Transverse Shear/Gage/Core
Average	1829.75	0.79	
St.dev.	96.52	0.05	

**Table 5:** Long beam flexural test—hybrid basalt/flax sandwich panel.

Specimen ID	Facing Stress (MPa)	Effective Facing Modulus (GPa)	$\mathrm{Flexural}\;\mathrm{Rigidity}\;(\mathrm{EI})\;(MNmm^{2})$	Flexural Strain at Failure (%)	Failure Mode
Basalt/Flax 4.1	28.77	8.83	36.93	0.31	Compressive skin delamination
Basalt/Flax 4.2	41.11	10.65	45.48	0.36	Compression
Basalt/Flax 4.3	38.92	10.14	44.48	0.36	Compression
Basalt/Flax 4.4	30.88	9.20	39.47	0.32	Tensile skin delamination
Basalt/Flax 4.5	35.37	9.96	42.17	0.33	Compressive skin delamination
Average	35.01	9.76	41.71	0.33	
St.dev.	5.21	0.73	3.53	0.02	

**Table 6:** Edgewise compression—hybrid basalt/flax sandwich.

Specimen ID	Compressive Facing Strength (MPa)	Effective Facing Modulus (GPa)	Failure Mode
Basalt/Flax 5.1	21.83	7.11	Facing Sheet Buckling
Basalt/Flax 5.2	27.84	17.60	Facing Sheet Buckling
Basalt/Flax 5.3	15.18	-	Facing Sheet Buckling
Basalt/Flax 5.4	28.53	13.25	Facing Sheet Buckling
Basalt/Flax 5.5	9.27	-	Facing Sheet Buckling
Average	20.53	12.65	
St.dev.	8.29	5.27	

**Table 7:** Core shear test—hybrid basalt/flax sandwich.

Specimen ID	Max Force (N)	Core Shear Ultimate Strength (GPa)	Failure Mode
Basalt/Flax 6.1	511.69	0.23	Skin to core delamination/Gage/Core-Facing Bond (DGA)
Basalt/Flax 6.2	746.07	0.34	DGA
Basalt/Flax 6.3	658.59	0.29	DGA
Basalt/Flax 6.4	749.99	0.33	DGA
Basalt/Flax 6.5	435.53	0.19	DGA
Average	620.37	0.28	
St.dev.	141.46	0.06	

**Table 8:** Comparison of reported flexural properties of bio-sandwich structures.

Skin	Core	Flexural Rigidity (D/b) (Nmm)	Reference
Jute/PP (2 mm)	PET foam (20 mm)	${465.19\times 10}^{3}$	[6]
UD flax/vinyl ester (1 layer)	Cork (11 mm)	$900.0\;{\times 10}^{3}$	[8]
UD flax/bio-epoxy (1 mm)	Corrugated cardboard (25 mm)	$4858.0\;{\times 10}^{3}$	[7]
White oak (1.59 mm)	Mushroom foam (25.4 mm)	$25.0\;{\times 10}^{3}$	[3]
Woven Glass fibre (0.24 mm)	PUR foam (32.36 mm)	$372.5\;{\times 10}^{3}$	[5]
Woven flax fibre (1.56 mm)	PIR foam (75 mm)	$54.0\;{\times 10}^{3}$	[4]
Non-woven Flax/bio-resin (1.4 mm)	PET foam (14 mm)	$925.1\;{\times 10}^{3}$	Present work
Woven basalt-flax/bio-resin (0.5 mm)	PET foam(14 mm)	$556.1\;{\times 10}^{3}$	Present work

## Data Availability

The original contributions presented in the study are included in the article, further inquiries can be directed to the corresponding authors.
